# ALL-CAUSE MORTALITY AND CAUSE OF DEATH IN US LONG-LIVED SIBLINGS: DATA FROM THE LONG LIFE FAMILY STUDY

**DOI:** 10.1093/geroni/igad104.1972

**Published:** 2023-12-21

**Authors:** Shanshan Yao, Robert Boudreau, Angéline Galvin, Joanne Murabito, Lawrence Honig, Kaare Christensen, Anne Newman

**Affiliations:** University of Pittsburgh School of Public Health, Pittsburgh, Pennsylvania, United States; University of Pittsburgh School of Public Health, Pittsburgh, Pennsylvania, United States; University of Southern Denmark, Odense, Syddanmark, Denmark; Boston University Chobanian & Avedisian School of Medicine, Boston, Massachusetts, United States; Columbia University, New York City, New York, United States; University of Southern Denmark, Odense, Syddanmark, Denmark; University of Pittsburgh School of Public Health, Pittsburgh, Pennsylvania, United States

## Abstract

This study compared the mortality risk of long-lived siblings with the general population in the US and investigated the leading causes of death and the correspondence in age at death and causes of death between long-lived siblings. In the Long Life Family Study (LLFS), 1,264 siblings (Mean age 90.1, SD 6.4) were recruited from the US and followed over 12 years. Their survival function, based on the Kaplan-Meier estimator, was compared with an age-, race-, and sex-matched sample from US census data. To examine underlying and contributing causes, we examined in detail 338 deaths with complete death adjudication using death certificates, family interview, hospital and nursing home records at the Pittsburgh field center through the year 2018. Concordance between siblings was examined using correlation coefficients, Bland-Altman plots, percent agreement, and Kappa statistics. The LLFS proband siblings had better survival compared with US general population matched on birth cohort and age at enrollment. Age at death ranged from 75-104, mean 91.4 (SD 4.8). The leading causes of death were cardiovascular disease (33.1%), dementia (22.2%), and cancer (10.7%). In addition, we did not find significant correspondence in the age at death, the underlying cause of death, or conditions directly contributing to death between siblings. Our findings demonstrate further exceptional longevity in the LLFS proband generation compared to the general population, but do not show significant correspondence in age at death and causes of death between siblings.

